# Multimodal ultrasound-based carotid plaque risk biomarkers predict poor functional outcome in patients with ischemic stroke or TIA

**DOI:** 10.1186/s12883-023-03052-6

**Published:** 2023-01-12

**Authors:** Linggang Cheng, Shuai Zheng, Jinghan Zhang, Fumin Wang, Xinyao Liu, Lin Zhang, Zhiguang Chen, Ye Cheng, Wei Zhang, Yi Li, Wen He

**Affiliations:** 1 Department of Ultrasound, Beijing Tiantan Hospital, Capital Medical University, No.119 South Fourth Ring West Road, Fengtai District, Beijing, 100160 China; 2 Guang’anmen Hospital, Chinese Academy of traditional Chinese Medicine, Beijing, China

**Keywords:** Multimodal ultrasound, Carotid plaque, Stroke, Shear wave elastography, Superb microvascular imaging

## Abstract

**Background:**

Carotid vulnerable plaque is an important risk factor for stroke occurrence and recurrence. However, the relationship between risk parameters related to carotid vulnerable plaque (plaque size, echogenicity, intraplaque neovascularization, and plaque stiffness) and neurological outcome after ischemic stroke or TIA is unclear. This study investigates the value of multimodal ultrasound-based carotid plaque risk biomarkers to predict poor short-term functional outcome after ischemic stroke or TIA.

**Methods:**

This study was a single-center, prospective, continuous, cohort study to observe the occurrence of adverse functional outcomes (mRS 2–6/3–6) 90 days after ischemic stroke or TIA in patients, where the exposure factors in this study were carotid plaque ultrasound risk biomarkers and the risk factors were sex, age, disease history, and medication history. Patients with ischemic stroke or TIA (mRS ≤3) whose ipsilateral internal carotid artery stenosis was ≥50% within 30 days were included. All patients underwent multimodal ultrasound at baseline, including conventional ultrasound, superb microvascular imaging (SMI), and shear wave elastography (SWE). Continuous variables were divided into four groups at interquartile spacing for inclusion in univariate and multifactorial analyses. After completion of a baseline ultrasound, all patients were followed up at 90 days after ultrasound, and patient modified neurological function scores (mRSs) were recorded. Multivariate Cox regression and ROC curves were used to assess the risk factors and predictive power for predicting poor neurological function.

**Results:**

SMI revealed that 20 (30.8%) patients showed extensive neovascularization in the carotid plaque, and 45 (69.2%) patients showed limited neovascularization in the carotid plaque. SWE imaging showed that the mean carotid plaque stiffness was 51.49 ± 18.34 kPa (23.19–111.39 kPa). After a mean follow-up of 90 ± 14 days, a total of 21 (32.3%) patients had a mRS of 2–6, and a total of 10 (15.4%) patients had a mRS of 3–6. Cox regression analysis showed that the level of intraplaque neovascularization and plaque stiffness were independent risk factors for a mRS of 2–6, and the level of intraplaque neovascularization was an independent risk factor for a mRS of 3–6. After correcting for confounders, the HR of intraplaque neovascularization level and plaque stiffness predicting a mRS 2–6 was 3.06 (95% CI 1.05–12.59, *P* = 0.041) and 0.51 (95% CI 0.31–0.83, *P* = 0.007), respectively; the HR of intraplaque neovascularization level predicting a mRS 3–6 was 6.11 (95% CI 1.19–31.45, *P* = 0.031). For ROC curve analysis, the mRSs for intraplaque neovascularization level, plaque stiffness, and combined application to predict 90-day neurological outcome ranged from 2 to 6, with AUCs of 0.73 (95% CI 0.59–0.87), 0.76 (95% CI 0.64–0.89) and 0.85 (95% CI 0.76–0.95), respectively. The mRSs for the intraplaque neovascularization level to predict 90-day neurological outcome ranged from 3 to 6, with AUCs of 0.79 (95% CI 0.63–0.95).

**Conclusion:**

Intraplaque neovascularization level and plaque stiffness may be associated with an increased risk of poor short-term functional outcome after stroke in patients with recent anterior circulation ischemic stroke due to carotid atherosclerosis. The combined application of multiple parameters has efficacy in predicting poor short-term functional outcome after stroke.

## Introduction

Inflammation, oxidative stress and vulnerable plaques play an important role in the development of acute ischemic cerebrovascular events [1–3]. Stress-related neurobiological activity is significantly associated with atherosclerotic plaque inflammation and instability through enhanced macrophage activity [4–6]. Studies have shown that vulnerable plaque is an independent risk factor for the development and recurrence of cardiovascular disease [7–11] and is significantly associated with increased mortality after stroke [12, 13].

Recent studies have shown that carotid plaque vulnerability biomarkers, plaque thickness, plaque size, plaque surface morphology and intraplaque neovascularization are associated with the occurrence and recurrence of ischemic cardiovascular events [14–17]. However, the relationship between carotid plaque vulnerability biomarkers (including plaque thickness, size, surface morphology, intraplaque neovascularization, and plaque stiffness) and neurological outcome after ischemic cerebrovascular events is unclear.

No studies have been reported on the association of carotid plaque vulnerability biomarkers with poor neurological outcome after ischemic cerebrovascular events. The purpose of this study was to apply multimodal ultrasound imaging techniques to assess the association of carotid plaque risk biomarkers with poor short-term neurological outcome after ischemic cerebrovascular events in a prospective cohort of patients with ischemic cerebrovascular events due to carotid atherosclerosis.

## Materials and methods

### Research subjects

Patients with ≥50% ipsilateral internal carotid artery stenosis confirmed by carotid ultrasound who had a recent (< 30 days) noncardiac ischemic cerebrovascular event (ischemic stroke, mRS ≤3 or TIA) were prospectively and continuously included in this study. All patients underwent bilateral carotid multimodality ultrasound at baseline, and the largest plaque causing carotid stenosis was included in the final analysis.

Inclusion criteria: 1. age ≥ 50 years; 2. anterior circulation ischemic ischemic stroke or TIA occurring within 30 days; 3. ipsilateral internal carotid artery stenosis ≥50%; and 4. mRS ≤ 3. Exclusion criteria: 1. history of neck radiotherapy; 2. diagnosis of cardiogenic stroke, small vessel strokes, stroke of unknown origin according to TOAST criteria; 3. carotid stenosis not due to atherosclerosis; 4. impaired consciousness to cooperate in completing ultrasound examination; 5. poor quality of ultrasound images; 6. history of carotid endarterectomy or stent placement; or 7. patient lost to follow-up. The study protocol was approved by the ethics committee, and all patients provided written informed consent before the ultrasound examination was performed.

### Ultrasonography

Images were acquired by one sonographer with more than 5 years of experience in vascular ultrasound and analyzed by two physicians with more than 5 years of experience in vascular ultrasound using a double-blind method. Ultrasound equipment (Canon Aplio 900, Canon Medical Systems Corporation, Japan) and high-frequency line array probes with frequencies of 5–14 MHz were used. The following ultrasound parameters were obtained at baseline using a multimodal ultrasound protocol: degree of carotid stenosis, length of stenotic segment, maximum plaque thickness, plaque size (area), plaque echogenicity, plaque surface morphology, plaque calcification, number of intraplaque neovascularizations, and plaque stiffness. The patient was supine, and his bilateral carotid arteries (including the common carotid artery, carotid bifurcation and internal carotid artery) were scanned in longitudinal, transverse and oblique views.

### Conventional ultrasound imaging

A carotid plaque was defined as a carotid artery with a local intima-media thickness greater than 1.5 mm or focal wall thickness greater than at least 50% of the surrounding vessel wall. Plaque echogenicity was classified according to grayscale ultrasound images: type 1, homogeneous hypoechoic plaque; type 2, hypoechoic predominant plaque; type 3, hypoechoic predominant plaque; and type 4, homogeneous hypoechoic plaque [18, 19]. Plaque ulceration was defined as the presence of an echogenic defect (at least 2 × 2 mm) on the plaque surface with colored flow signal filling visible on CDFI. The degree of internal carotid artery stenosis was classified as 50–69% and 70–99% with reference to peak systolic flow velocity and end-diastolic flow velocity, respectively [20].

### SMI

After conventional ultrasonography, the SMI mode was activated to display the target plaque in grayscale mode and monochrome SMI mode in dual format in real time. The SMI specific region of interest box adjustment included the plaque. The SMI scan parameters were set as follows: mechanical index 1.5, frame rate 50–60 fps, dynamic range 55–60 dB, and speed range 1.0–2.0 cm/s. The SMI video images of the plaque of interest were stored in transverse and longitudinal sections for 1 minute each. The intraplaque dynamic enhancement signal was defined as intraplaque microvascular flow (IMVF), and static enhancement was considered an artifact. The IMVF class was classified according to the following visual scale: 1. no IMVF or IMVF confined to the extravascular membrane; 2. dynamic IMVF reaching the shoulder of the plaque; 3. IMVF reaching the plaque core; and 4. extensive neovascularization within the plaque. IPN was classified as limited (IMVF level 1 or 2) and extensive (IMVF level 3 or 4) according to the IMVF level.

### SWE imaging

SWE software was enabled after SMI examination, and the elasticity map and quality control map of the plaque of interest were displayed in dual-amplitude real-time. Young’s modulus (YM, kPa) was calculated according to the shear wave propagation equation: YM = ρc2, where YM is the tissue elasticity, ρ is the tissue density, and c is the shear wave velocity. SWE parameter settings were as follows: resolution = 3; smoothing = 3; FR control = 3; and focus = 75%. The SWE region of interest sampling frame was adjusted to include the entire plaque. During the SWE examination, the probe was placed lightly on the surface of the skin to reduce the effect of pressure on the measurement results, and the elastography imaging was stabilized for approximately 3 seconds for the elasticity measurement. Plaque elasticity measurements were performed by manually outlining the entire plaque to measure the overall elasticity of the plaque. Each plaque was measured three times and averaged. The elastography system also generates a quality control map, and measurements are more reliable and accurate when the propagation lines are parallel to each other; otherwise, if the propagation lines are distorted or missing, a new measurement is needed.

### Clinical variables

The following variables were collected by a neurologist using a blinded method: (1) age and sex; (2) past medical history, including hypertension, diabetes mellitus, dyslipidemia, history of ischemic cerebrovascular events and coronary artery disease; (3) history of smoking and alcohol consumption; (4) history of drug use; (5) height, weight and body mass index; (6) modified Rankin score (mRS) after stroke; and (7) etiology of ischemic cerebrovascular events.

### Ending events and follow-up

After completion of baseline ultrasound, all patients were followed up at a standardized 90-day post ultrasound visit to assess their neurological function (mRS) status after the occurrence of an ischemic cerebrovascular event. Neurological function score assessment was determined by microphone or telephone interview and confirmed by reviewing hospital records. Neurological function outcomes included a 90-day mRS of 2–6/3–6. Clinical events were determined, and ultrasound image results were interpreted by a blinded method.

### Statistical analysis

All statistical analyses were performed using SPSS 26.0 statistical analysis software (IBM Corporation, New York, USA). Continuous variables are expressed as the mean ± standard deviation, and categorical variables are expressed as frequencies or percentages. Patients were divided into four groups according to plaque hardness for baseline comparisons. The nonparametric Wilcoxon or Kruskal–Wallis test was used for continuous variables, and the χ^2^ test was used for categorical variables. Continuous variables were included in the regression model in the form of quartile spacing. Cox proportional risk regression was used to analyze the association between carotid plaque risk biomarkers and adverse functional outcomes. Variables with a univariate analysis of *P* < 0.1 and clinically accepted predictive parameters were included as confounding variables in the multifactorial model. Uncorrected and corrected hazard ratios (HRs) and 95% confidence intervals (CIs) were calculated. The predictive efficacy of the model was evaluated by applying the subject operating characteristic (ROC) curve and the area under the curve (AUC). *P* < 0.05 was defined as a statistically significant difference.

## Results

### Baseline patient characteristics

From July 2019 to February 2020, 73 patients with symptomatic carotid stenosis were included, of whom 6 underwent carotid revascularization and 2 lost-to-review patients were excluded. Finally, 65 patients with complete clinical and ultrasound data were included in the final analysis. These included 48 (73.8%) males with a mean age of 67.9 ± 7.7 years. Thirty-six (55.4%) had hypertension, 17 (26.2%) had diabetes mellitus, and 32 (49.2%) had dyslipidemia. The baseline data of the enrolled patients are shown in Table 1. Seventeen (26.2%) of these patients had moderate carotid stenosis, and 48 (73.8%) had severe carotid stenosis. The number of stroke was 35, and the number of TIA was 30 in our study. The average ABCD2 score was 3.62 ± 1.32. The average mRS of patients enrolled was 1.31 ± 1.21. All of patients is the laterality of the strokes the same as the side as the laterality of the carotid disease. There were no patient deaths during the study.

**Table 1:** Baseline characteristics of included patients: grouped according to quartiles of plaque elasticity (*n* = 65)

Variables	Quartiles of plaque elasticity				P
	Quartile 1	Quartile 2	Quartile 3	Quartile 4	
Age, years	67.7 ± 7.5	67.3 ± 8.2	66.7 ± 8.2	70.1 ± 7.3	0.629
Sex (male)	64.7%(11)	68.8%(11)	68.8%(11)	93.8%(15)	0.177
Body mass index，kg/m^2^	25.88 ± 2.39	23.49 ± 1.09	24.04 ± 1.06	23.58 ± 2.41	0.004
Smoking	76.5%(13)	43.8%(7)	81.3%(13)	75.0%(12)	0.109
Drinking alcohol	70.6%(12)	68.8%(11)	56.3%(9)	87.5%(14)	0.306
SBP，mmHg	143.8 ± 20.2	144.2 ± 28.2	142.1 ± 23.7	140.4 ± 18.8	0.927
DBP，mmHg	82.8 ± 7.0	83.8 ± 13.2	85.6 ± 10.1	86.1 ± 6.9	0.388
Hypertension	64.7%(11)	56.3%(9)	56.3%(9)	43.8%(7)	0.716
Diabetes mellitus	17.6%(3)	62.5%(10)	18.8%(3)	6.3%(1)	0.002
Dyslipidemia	52.9%(9)	50.0%(8)	62.5%(10)	31.3%(5)	0.373
Coronary heart disease	52.9%(9)	25.0%(4)	31.3%(5)	18.8%(3)	0.179
Anti-platelet drugs	88.2%(15)	75.0%(12)	75.0%(12)	75.0%(12)	0.734
Anti-hypertensive	70.6%(12)	62.5%(10)	68.8%(11)	56.3%(9)	0.827
Statin	76.5%(13)	93.8%(15)	93.8%(15)	75.0%(12)	0.262
Degree of stenosis					0.341
50–69%	17.6%(3)	25.0%(4)	43.8%(7)	18.8%(3)	
70–99%	82.4%(14)	75.0%(12)	56.3%(9)	81.3%(13)	
Plaque thickness，(cm)	0.37 ± 0.07	0.39 ± 0.06	0.35 ± 0.06	0.39 ± 0.09	0.146
Plaque area，(cm^2^)	0.58 ± 0.11	0.69 ± 0.18	0.63 ± 0.25	0.69 ± 0.17	0.196
Plaque length，(cm)	1.8 ± 0.43	2.02 ± 0.47	1.81 ± 0.51	2.13 ± 0.39	0.089
Calcified plaque	41.2%(7)	62.5%(10)	31.3%(5)	81.3%(13)	0.021
Plaque echogenicity					0.112
Homogeneous hyperechogenicity	5.9%(1)	6.3%(1)	18.8%(3)	6.3%(1)	
predominantly low echogenicity	58.8%(10)	50.0%(8)	56.3%(9)	31.3%(5)	
predominantly high echogenicity	11.8%(2)	43.8%(7)	12.5%(2)	31.3%(5)	
Homogeneous high echogenicity	23.5%(4)	0	12.5%(2)	31.3%(5)	
Plaque surface morphology					0.115
Smooth	35.3%(6)	18.8%(3)	6.3%(1)	31.3%(5)	
Irregular	35.3%(6)	56.3%(9)	87.5%(14)	56.3%(9)	
Ulcers	29.4%(5)	25.0%(4)	6.3%(1)	12.5%(2)	
Neovascularization level					0.533
Restricted	70.6%(12)	56.3%(9)	68.8%(11)	81.3%(13)	
Extensive	29.4%(5)	43.8%(7)	31.3%(5)	18.8%(3)	

### Plaque characteristics

The average thickness of plaques was 0.38 ± 0.07 cm, the average length of plaques was 1.94 ± 0.46 cm, and the average area of plaques was 0.65 ± 0.19 cm^2^. The proportions of uniform hypoechoic plaques, hypoechoic predominant plaques, hyperechoic predominant plaques and uniform hyperechoic plaques were 9.2% (6), 49.2% (32), 24.6% (16) and 16.9% (11), respectively. Calcified plaques accounted for 53.8% (35), and noncalcified plaques accounted for 46.2% (30). Of the 65 plaques, 45 (69.2%) showed limited IMVF on SMI, and 20 (30.8%) showed diffuse IMVF on SMI. The mean YM of the plaques was 51.50 ± 18.34 kPa (23.19 ~ 111.39 kPa). The baseline characteristics of the plaques are shown in Table 1.

### Primary outcome follow-up

A total of 21 patients (32.3%) had a poor functional outcome (mRS 2–6) at 90 days, and 10 patients (15.4%) had a mRS of 3–6 at 90 days. The mean time interval between baseline ultrasonography and functional outcome assessment was 90 ± 14 days in all patients.

### Analysis of risk factors associated with poor neurological outcomes

**Fig. 1:**
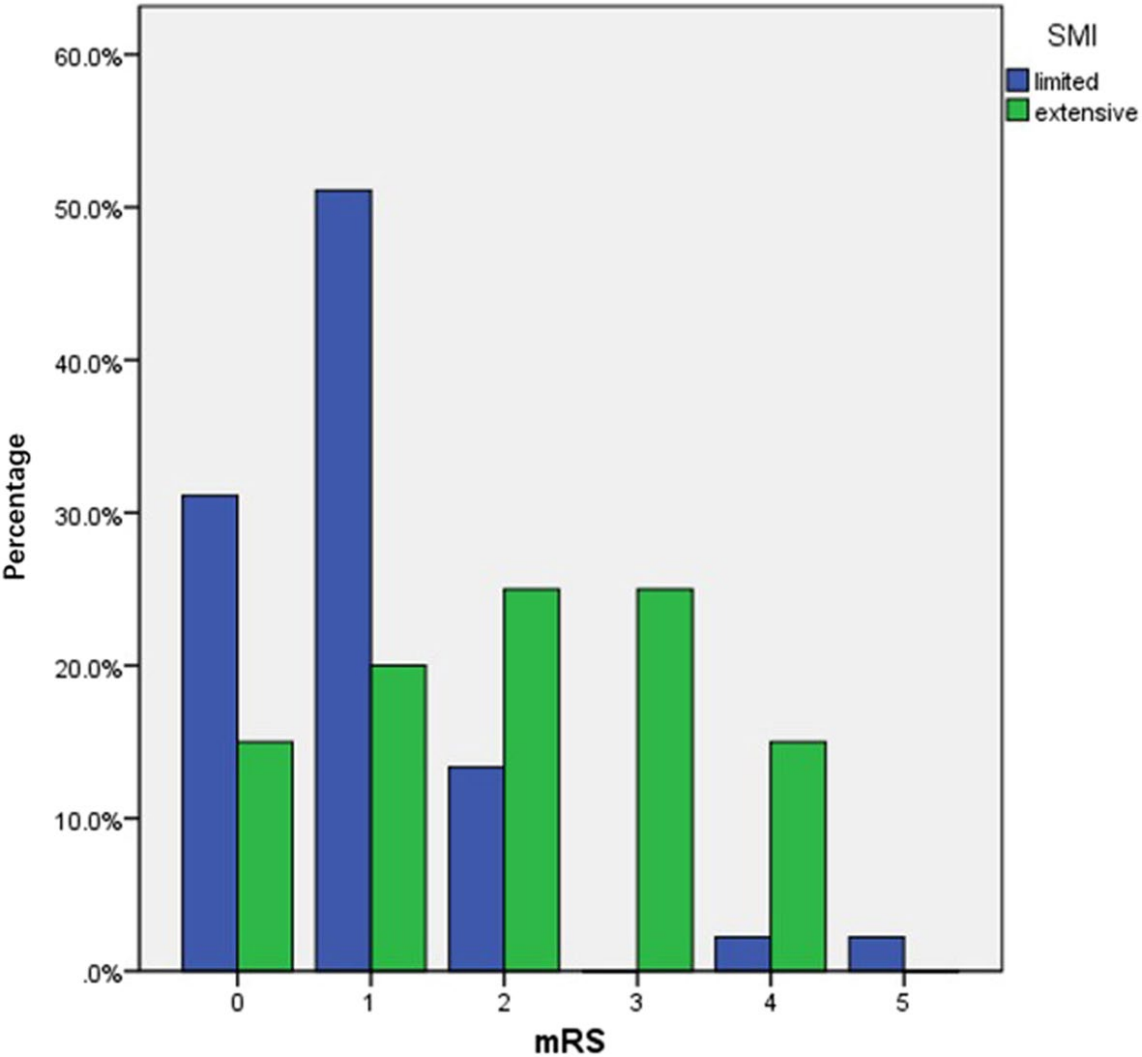
Images showing the distribution of neurological function scores after 90 days for different grades (limited, extensive) of the amount of neovascularization within the plaque

**Fig. 2:**
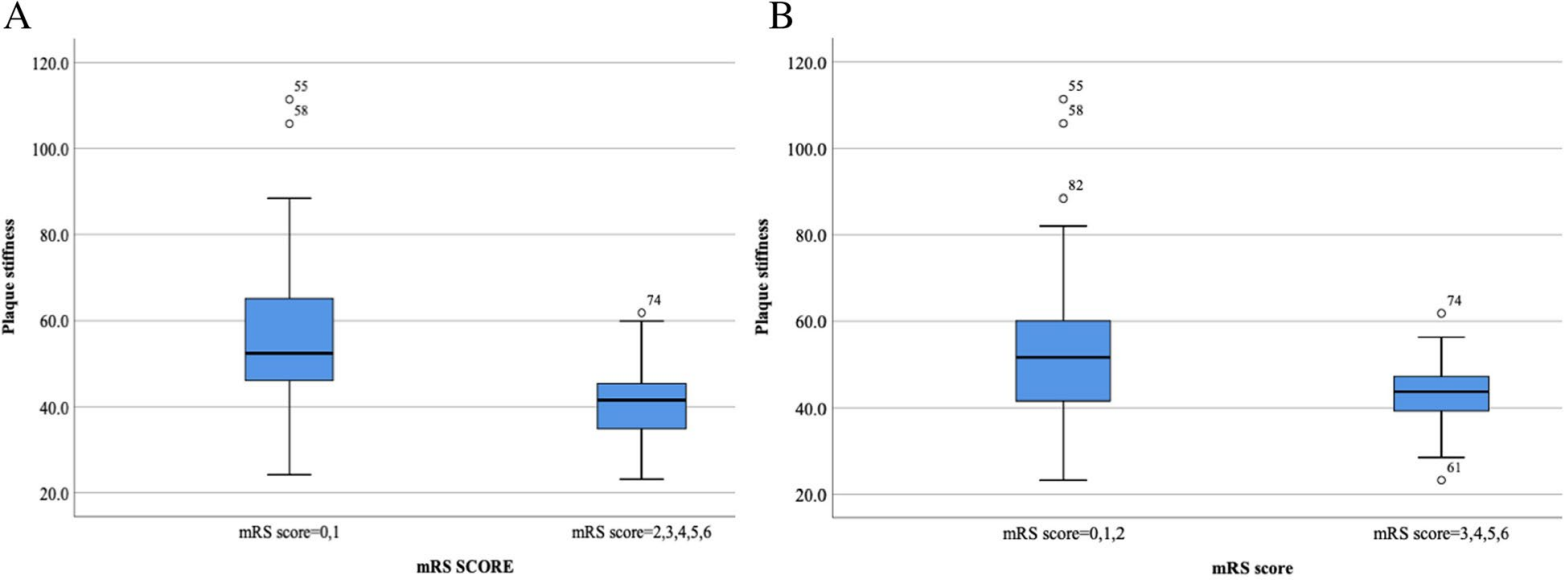
Images showing plaque stiffness distribution for 90-day neurofunctional prognosis

The association of carotid plaque ultrasound risk biomarkers with poor functional outcome after the onset of ischemic cerebrovascular time is shown in Table 2. After correction for potential confounders (age, sex, body mass index, history of smoking, alcohol consumption, history of concomitant disease, and history of drug use), patients with higher levels of neovascularization within the carotid plaque (extensive) were associated with an increased risk of poor functional outcome 90 days after stroke. The distribution of neurological function scores after 90 days for different grades (limited, extensive) of intraplaque neovascularization levels is shown in Fig. 1 (*P* < 0.001). The HR for intraplaque neovascularization level and a 90-day poststroke mRS of 2–6/3–6 was 3.64 (95% CI 1.05–12.59, *P* = 0.041) and 6.11 (95% CI 1.19–31.45, *P* = 0.031), respectively. Figure 2A shows the relationship between a 90-day poststroke mRS of 2–6 and plaque stiffness (*P* = 0.003), and Fig. 2B shows the relationship between a 90-day poststroke mRS of 3–6 and plaque stiffness (*P* = 0.119). Carotid plaque stiffness was associated with an increased risk of a mRS of 2–6 at 90 days poststroke, and the HR between plaque stiffness and a mRS of 2–6 at 90 days poststroke was 0.51 (95% CI 0.31–0.83, *P* = 0.007) after correcting for potential confounders. Multifactorial regression analysis showed that the level of intraplaque neovascularization was associated with an increased risk of poor functional outcome (2–6/3–6) 90 days after stroke (Table 3). The HRs for intraplaque neovascularization level and a mRS of 2–6/3–6 at 90 days poststroke were 3.35 (95% CI 1.39–8.09, *P* = 0.007) and 6.68 (95% CI 1.37–34.33, *P* = 0.019), respectively. Plaque stiffness was associated with an increased risk of a mRS of 2–6 at 90 days poststroke, and the HR between plaque stiffness and a mRS of 2–6 at 90 days poststroke was 0.54 (95% CI 0.33–0.86, *P* = 0.009).

**Table 2:** Predictors of poor neurological outcome: univariate Cox analysis

outcomes	mRS score 2–6			mRS score 3–6		
Variables	HR	95%CI	P	HR	95%CI	P
Age, years	1.17	0.79–1.71	0.438	1.97	1.04–3.75	0.039
Sex (male)	1.51	0.51–4.47	0.462	1.42	0.30–6.67	0.660
Body mass index, kg/m2	1.22	0.83–1.79	0.306	1.03	0.59–1.79	0.914
Smoking	1.11	0.43–2.86	0.827	1.78	0.38–8.37	0.467
Drinking alcohol	1.03	0.41–2.66	0.947	0.96	0.25–3.73	0.957
Hypertension	1.61	0.65–3.99	0.303	1.21	0.34–4.28	0.769
Diabetes mellitus	1.74	0.72–4.19	0.219	1.88	0.53–6.67	0.327
Dyslipidemia	0.78	0.33–1.84	0.560	0.44	0.11–1.71	0.237
Coronary heart disease	1.29	0.53–3.11	0.572	2.09	0.61–7.24	0.242
Anti-platelet drugs	0.82	0.24–2.85	0.759	0.34	0.08–1.41	0.333
Anti-hypertensive	0.62	0.21–1.82	0.386	0.30	0.07–1.20	0.088
Statin	0.67	0.18–2.69	0.573	0.34	0.07–1.63	0.178
Degree of stenosis	1.13	0.42–3.09	0.807	0.53	0.15–1.88	0.327
Plaque thickness，(cm)	0.92	0.62–1.36	0.682	0.72	0.39–1.30	0.278
Plaque area，(cm^2^)	0.92	0.63–1.34	0.650	1.04	0.61–1.79	0.881
Plaque length，(cm)	1.11	0.75–1.63	0.605	1.59	0.88–2.91	0.125
Calcified plaque	0.64	0.27–1.53	0.316	0.86	0.25–2.96	0.807
Echo of plaque	0.71	0.42–1.19	0.188	0.57	0.26–1.26	0.163
Plaque surface morphology	1.12	0.58–2.17	0.742	1.42	0.54–3.75	0.473
Neovascularization level	3.66	1.52–8.82	0.004	9.0	1.91–42.38	0.005
Plaque hardness	0.53	0.34–0.83	0.006	0.61	0.33–1.13	0.117

**Table 3:** Predictors of poor neurological outcome: multifactorial Cox analysis

outcomes	mRS score 2–6			mRS score 3–6		
Variables	HR	95%CI	P	HR	95%CI	P
Model 1						
Neovascularization level	3.35	1.39–8.09	0.007	6.86	1.37–34.33	0.019
Plaque stiffness	0.54	0.33–0.86	0.009	/	/	/
Model 2						
Neovascularization level	3.06	1.19–7.79	0.019	6.99	1.38–35.51	0.019
Plaque stiffness	0.53	0.33–0.84	0.007	/	/	/
Model 3						
Neovascularization level	3.64	1.05–12.59	0.041	6.11	1.19–31.45	0.031
Plaque stiffness	0.51	0.31–0.83	0.007	/	/	/

Model 1: uncorrected for confounders; Model 2: corrected for age and gender; Model 3: corrected for age, gender, body mass index, history of smoking, alcohol consumption, history of concomitant disease, and history of drug use

### ROC curve analysis

**Fig. 3:**
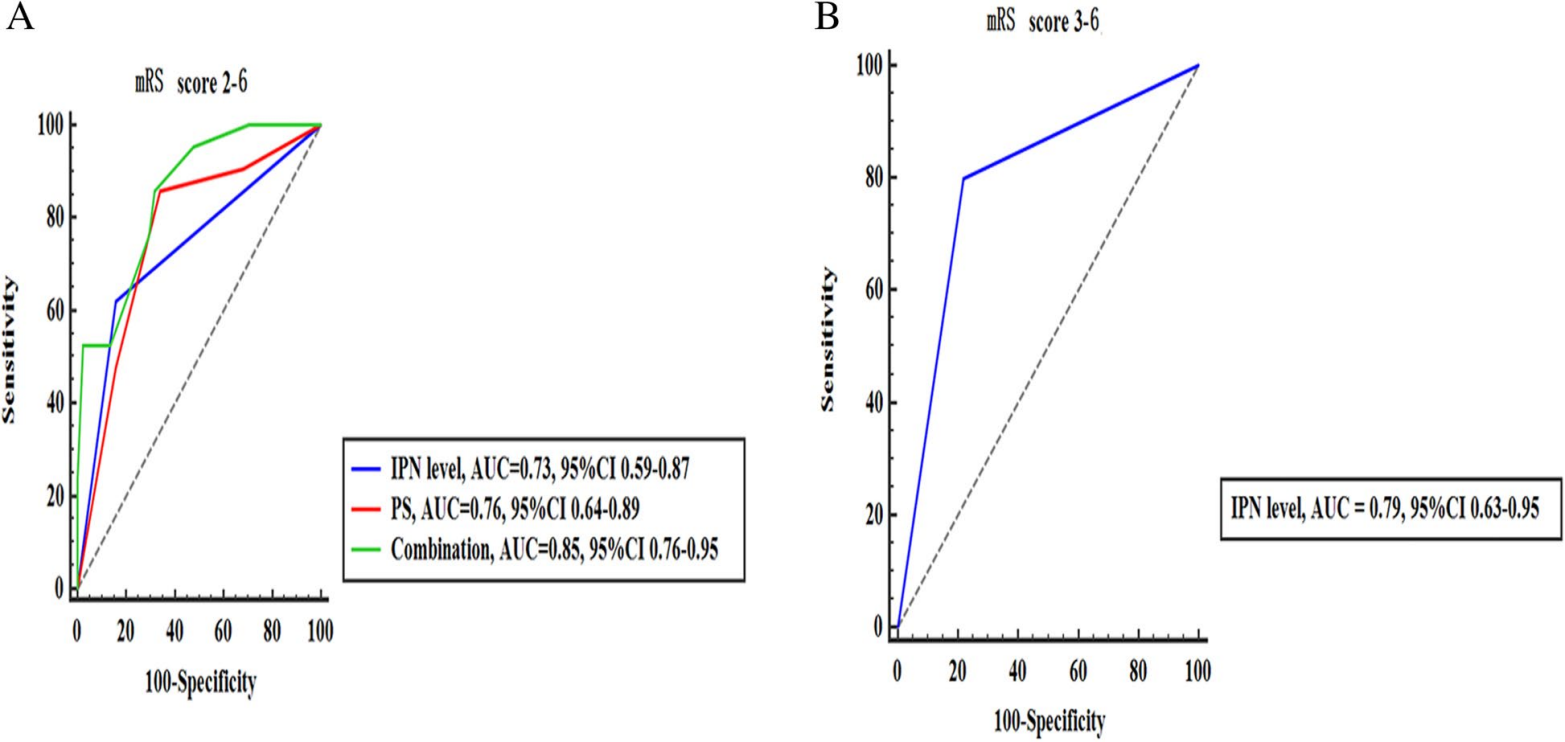
ROC curve analysis. A. Intraplaque neovascularization level, plaque stiffness and a combination of both predicted 90-day neurological outcome mRS score 2 to 6: IPN grade (blue line, AUC = 0.73), plaque stiffness (red line, AUC = 0.76) and combined application (green line, AUC = 0.85) ROC curves. The highest identification accuracy was achieved with the combined application, AUC = 0.85. B. Intraplaque neovascularization level predicts 90-day neurological outcome mRS score 3 to 6 with AUC 0.79. IPN = intraplaque neovascularization, PS = plaque hardness, AUC = area under the ROC curve, CI = confidence interval

The ROC curve analysis of intraplaque neovascularization level, plaque stiffness and combined application to predict poor functional outcome (mRS 2–6) 90 days after stroke is shown in Fig. 3A. The highest predictive accuracy of the combined application of multiple parameters was AUC = 0.85, 95% CI 0.76–0.95. The Delong test showed that the accuracy of the differential diagnosis of the combined application of the multiple parameter model was significantly higher than that of the single parameter model (all *P* < 0.05). The sensitivity, specificity, positive predictive value, negative predictive value, and diagnostic accuracy of the combined application of multiple parameters to predict poor functional outcome 90 days after stroke were 81.1, 84.6%，70.8, 90.2 and 83.1%, respectively. The ROC curve analysis of intraplaque neovascularization level to predict a mRS of 3–6 90 days after stroke is shown in Fig. 3B. The AUC of the intraplaque neovascularization level to predict a mRS of 3–6 90 days after stroke was 0.79 (95% CI 0.63–0.95). The accuracy of the intraplaque neovascularization level to predict a mRS of 3–6 90 days after stroke was 84.6%.

## Discussion

This study investigated the relationship between carotid plaque risk biomarkers and short-term functional outcome after ischemic cerebrovascular events in patients with atherosclerosis. The results of the study showed that carotid intraplaque neovascularization level was an independent predictor of poor short-term functional outcome after stroke (mRS of 2–6/3–6). The accuracy of the carotid intraplaque neovascularization level in predicting poor short-term functional outcome after stroke was high. In addition, our study also found that carotid plaque stiffness was an independent risk factor for poor short-term functional outcome after stroke (mRS 3–6).

Carotid vulnerable plaque is an important risk factor for stroke onset and recurrence [21, 22]. In addition, carotid vulnerable plaque is associated with the effects of oxidative stress and inflammation [23–25]. Currently, there are few studies related to the relationship between carotid vulnerable plaque and stroke prognosis. Guo et al. [26] found that combined inflammatory and atherosclerotic biomarkers were effective in predicting 90-day prognosis after ischemic stroke and provided additional information on the burden of poor functional outcome in patients with ischemic stroke. Another study has shown that statin use in patients with acute large atherosclerotic stroke is associated with a reduction in stroke-inducing microembolic signals and a lower microembolic burden, resulting in improved neurological outcomes in patients [27]. Our study confirms and extends previous studies. The level of neovascularization and plaque hardness in carotid plaques can evaluate vulnerable plaques and reflect the severity of carotid atherosclerosis, which has value in predicting the prognosis of ischemic cerebrovascular events.

Previous studies have shown that vulnerable plaques (level of intraplaque neovascularization and plaque stiffness, etc.) are associated with the occurrence and recurrence of ischemic cerebrovascular events [28, 29]. However, no studies have examined the relationship between carotid vulnerable plaque risk biomarkers and functional outcome in stroke. Our findings suggest that intraplaque neovascularization levels and plaque stiffness are associated with an increased risk of poor functional outcome after stroke. The mechanisms that correlate carotid vulnerable plaque risk biomarkers with the prognosis of functional outcome in stroke are unclear. Potential relevant mechanisms are as follows: first, the development of carotid vulnerable plaque is associated with processes such as inflammation and oxidative stress, which can damage vascular endothelial cells, form foam cells, and activate cell surface receptors on monocytes and vascular smooth muscle cells, thereby initiating the inflammatory process and promoting the progression of atherosclerosis (plaque) [30–32]. In addition, microembolic dislodgement caused by vulnerable carotid plaques is an independent risk factor for stroke progression, neurological impairment and poor prognosis in patients with acute ischemic stroke [33]. Therefore, carotid plaque risk biomarkers may be an independent risk factor for poor functional outcome in patients with mini-stroke and transient ischemic attack.

The strengths of this study are that it is a prospective cohort study that confirms for the first time that carotid plaque risk biomarkers (intraplaque neovascularization level and plaque stiffness) are independent risk factors for poor short-term neurological outcome after ischemic cerebrovascular events. This study also has the following limitations: this study is a single-center study with a relatively small sample of included studies, and further validation in a multicenter, large sample study is needed for follow-up. The follow-up period of this study was short, and the neurological functional outcome may change as the follow-up period increases. In addition, only the ultrasound characteristics of larger plaques at the stenosis were evaluated in this study, and the ultrasound characteristics of the remaining plaques were not evaluated, which may have missed information on plaques that have an impact on functional outcome. The prior presence of a TIA is associated with a good early outcome in non-lacunar ischemic strokes, thus suggesting a neuroprotective effect of TIA possibly by inducing a phenomenon of ischemic tolerance [34]. This may be a factor affecting the prognosis of patients. We did not accounted the baseline ischemic stroke or TIA characteristics and the distribution of stroke vs TIA patients in our study, we will further analyze this issue in the next study. Finally, the exclusion of severely calcified carotid plaques from this study may have introduced a selection bias in the results of the analysis.

## Conclusion

Our study shows that carotid plaque risk biomarkers (intraplaque neovascularization level and plaque stiffness) may be associated with an increased risk of poor short-term functional outcome after stroke in patients with recent anterior circulation ischemic stroke due to carotid atherosclerosis. The combined application of multiple parameters has efficacy in predicting poor short-term functional outcome after stroke. These parameters may alter clinical management decisions in stroke patients, but further validation of the predictive value of these parameters in a large population and multicenter studies is needed.

## Data Availability

The datasets generated and/or analyzed during the current study are not publicly available due to multidisciplinary cooperation of our hospital, but are available from the corresponding author on reasonable request.

## References

[1] Hansson GK (2005). Inflammation, atherosclerosis, and coronary artery disease. N Engl J Med.

[2] Allen CL, Bayraktutan U (2009). Oxidative stress and its role in the pathogenesis of ischaemic stroke. Int J Stroke.

[3] Kamtchum-Tatuene J, Noubiap JJ, Wilman AH (2020). Prevalence of high-risk plaques and risk of stroke in patients with asymptomatic carotid stenosis: a Meta-analysis. JAMA Neurol.

[4] Kang DO, Eo JS, Park EJ (2021). Stress-associated neurobiological activity is linked with acute plaque instability via enhanced macrophage activity: a prospective serial 18F-FDG-PET/CT imaging assessment. Eur Heart J.

[5] Fang R, Zhang N, Wang C (2011). Relations between plasma ox-LDL and carotid plaque among Chinese Han ethnic group. Neurol Res.

[6] Pirillo A, Norata GD, Catapano AL (2013). LOX-1, OxLDL, and atherosclerosis. Mediat Inflamm.

[7] Kelly PJ, Camps-Renom P, Giannotti N (2019). Carotid plaque inflammation imaged by 18F-Fluorodeoxyglucose positron emission tomography and risk of early recurrent stroke. Stroke.

[8] Ospel JM, Singh N, Marko M (2020). Prevalence of ipsilateral nonstenotic carotid plaques on computed tomography angiography in embolic stroke of undetermined source. Stroke.

[9] Gardener H, Caunca MR, Dong C (2017). Ultrasound markers of carotid atherosclerosis and cognition: the northern Manhattan study. Stroke.

[10] Selwaness M, Bos D, van den Bouwhuijsen Q (2016). Carotid atherosclerotic plaque characteristics on magnetic resonance imaging relate with history of stroke and coronary heart disease. Stroke.

[11] Zavodni AE, Wasserman BA, McClelland RL (2014). Carotid artery plaque morphology and composition in relation to incident cardiovascular events: the multi-ethnic study of atherosclerosis (MESA). Radiology.

[12] Fleg JL, Stone GW, Fayad ZA (2012). Detection of high-risk atherosclerotic plaque: report of the NHLBI working group on current status and future directions. JACC Cardiovasc Imaging.

[13] Eldrup N, Grønholdt ML, Sillesen H (2006). Elevated matrix metalloproteinase-9 associated with stroke or cardiovascular death in patients with carotid stenosis. Circulation.

[14] Barnett HJ, Taylor DW, Eliasziw M (1998). Benefit of carotid endarterectomy in patients with symptomatic moderate or severe stenosis. North American symptomatic carotid endarterectomy trial collaborators. N Engl J Med.

[15] Camps-Renom P, Prats-Sánchez L, Casoni F (2020). Plaque neovascularization detected with contrast-enhanced ultrasound predicts ischaemic stroke recurrence in patients with carotid atherosclerosis. Eur J Neurol.

[16] Zhao X, Hippe DS, Li R (2017). Prevalence and characteristics of carotid artery high-risk atherosclerotic plaques in Chinese patients with cerebrovascular symptoms: a Chinese atherosclerosis risk evaluation II study. J Am Heart Assoc.

[17] Lu M, Peng P, Cui Y (2018). Association of progression of carotid Artery Wall volume and recurrent transient ischemic attack or stroke: a magnetic resonance imaging study. Stroke.

[18] Arnold JA, Modaresi KB, Thomas N (1999). Carotid plaque characterization by duplex scanning: observer error may undermine current clinical trials. Stroke.

[19] Gray-Weale AC, Graham JC, Burnett JR (1988). Carotid artery atheroma: comparison of preoperative B-mode ultrasound appearance with carotid endarterectomy specimen pathology. J Cardiovasc Surg.

[20] Grant EG, Benson CB, Moneta GL (2003). Carotid artery stenosis: grayscale and doppler ultrasound diagnosis-society of radiologists in ultrasound consensus conference. Ultrasound Q.

[21] Schindler A, Schinner R, Altaf N, Hosseini AA (2020). Prediction of stroke risk by detection of hemorrhage in carotid plaques: Meta-analysis of individual patient data. JACC Cardiovasc Imaging.

[22] Sun J, Zhao XQ, Balu N (2017). Carotid plaque lipid content and fibrous cap status predict systemic CV outcomes: the MRI substudy in AIM-HIGH. JACC Cardiovasc Imaging.

[23] Orion D, von Landenberg P, Itsekson-Hayosh Z (2020). Plasma myeloperoxidase levels in acute brain ischaemia and high-grade carotid stenosis. Eur J Neurol.

[24] Markstad H, Edsfeldt A, Yao Mattison I (2019). High levels of soluble Lectinlike oxidized low-density lipoprotein Receptor-1 are associated with carotid plaque inflammation and increased risk of ischemic stroke. J Am Heart Assoc.

[25] Tomaniak M, Katagiri Y, Modolo R (2020). Vulnerable plaques and patients: state-of-the-art. Eur Heart J.

[26] Guo D, Zhu Z, Zhong C, Wang A (2020). Prognostic metrics associated with inflammation and atherosclerosis signaling evaluate the burden of adverse clinical outcomes in ischemic stroke patients. Clin Chem.

[27] Safouris A, Krogias C, Sharma VK (2017). Statin pretreatment and microembolic signals in large artery atherosclerosis. Arterioscler Thromb Vasc Biol.

[28] Di Leo N, Venturini L, de Soccio V (2018). Multiparametric ultrasound evaluation with CEUS and shear wave elastography for carotid plaque risk stratification. J Ultrasound.

[29] Song Y, Dang Y, Wang J (2021). Carotid Intraplaque neovascularization predicts ischemic stroke recurrence in patients with carotid atherosclerosis. Gerontology.

[30] Steinberg D, Lewis A (1997). Conner Memorial Lecture. Oxidative modification of LDL and atherogenesis. Circulation.

[31] Boullier A, Bird DA, Chang MK (2001). Scavenger receptors, oxidized LDL, and atherosclerosis. Ann N Y Acad Sci.

[32] Zhang XG, Xue J, Yang WH (2021). Inflammatory markers as independent predictors for stroke outcomes. Brain Behave.

[33] Spence JD (2015). Management of asymptomatic carotid stenosis. Neurol Clin.

[34] Arboix A, Cabeza N, Garcia-Eroles L (2004). Relevance of transient ischemic attack to early neurological recovery after nonlacunar ischemic stroke. Cerebrovasc Dis.

